# Supplementary material for: STRT-seq-2i: dual-index 5ʹ single cell and nucleus RNA-seq on an addressable microwell array
Source: Sci Rep. 2017 Nov 27;7:16327. doi: 10.1038/s41598-017-16546-4 (PMC5703850; doi:10.1038/s41598-017-16546-4)
Supplement: Supplementary file 1 — Supplementary Information [file 41598_2017_16546_MOESM1_ESM.pdf]

Supplementary Information

STRT-seq-2i: dual-index 5' single cell and nucleus RNA-seq on an addressable microwell array

Hannah Hochgerner[1,2], Peter Lönnerberg[1,2], Rebecca Hodge[3], Jaromir Mikes[2], Abeer Heskol[1], Hermann Hubschle[4], Philip Lin[4], Simone Picelli[1,2], Gioele La Manno[1,2], Michael Ratz[5], Jude Dunne[4], Syed Husain[4], Ed Lein[3], Maithreyan Srinivasan[4], Amit Zeisel[1,2]† and Sten Linnarsson[1,2]†

[1] Division of Molecular Neurobiology, Dept. of Medical Biochemistry and Biophysics, Karolinska Institutet, Stockholm, Sweden

[2] Science for Life Laboratory, Solna, Sweden

[3] Allen Institute for Brain Science, Seattle, Washington, USA

[4] WaferGen Biosystems Inc., Fremont, California, USA

[5] Dept. of Cell and Molecular Biology, Karolinska Institutet, Stockholm, Sweden

† Corresponding authors. Email: amit.zeisel@ki.se (A.Z.) sten.linnarsson@ki.se (S.L.)

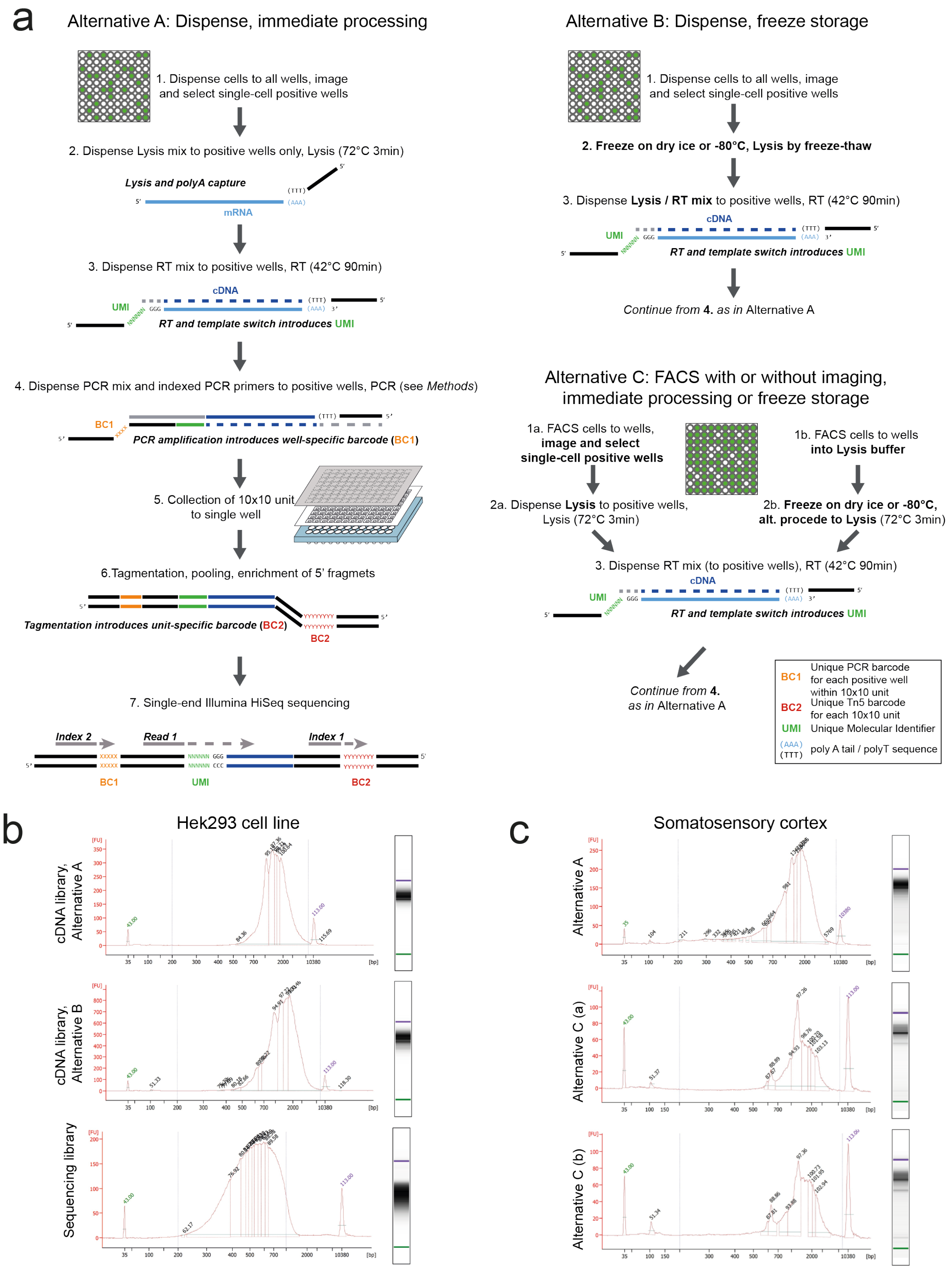

Figure S1

(a) Overview of three different STRT-seq-2i workflows: immediate processing (Alternative A), freeze storage (Alternative B) or FACS dispense, with or without freezing (Alternative C). A 6 bp UMI is introduced by template switching in the reverse transcription (RT) reaction. The first PCR incorporates one index on the plate, and after extraction the second index is introduced by tagmentation. Sequencing libraries are single-end read, with 48bp read 1, and two index reads (8 and 5 bp). (b) BioAnalyzer traces of Hek293 cell cDNA libraries generated from one subarray (20-30 cells) each, using Alternative A or B as indicated, and the final ready

sequencing library from one full plate. (c) cDNA libraries of cortex cells dispensed (Alternative A) or FACS sorted with or without imaging (Alternative C), from one unit (15-30 cells) each.

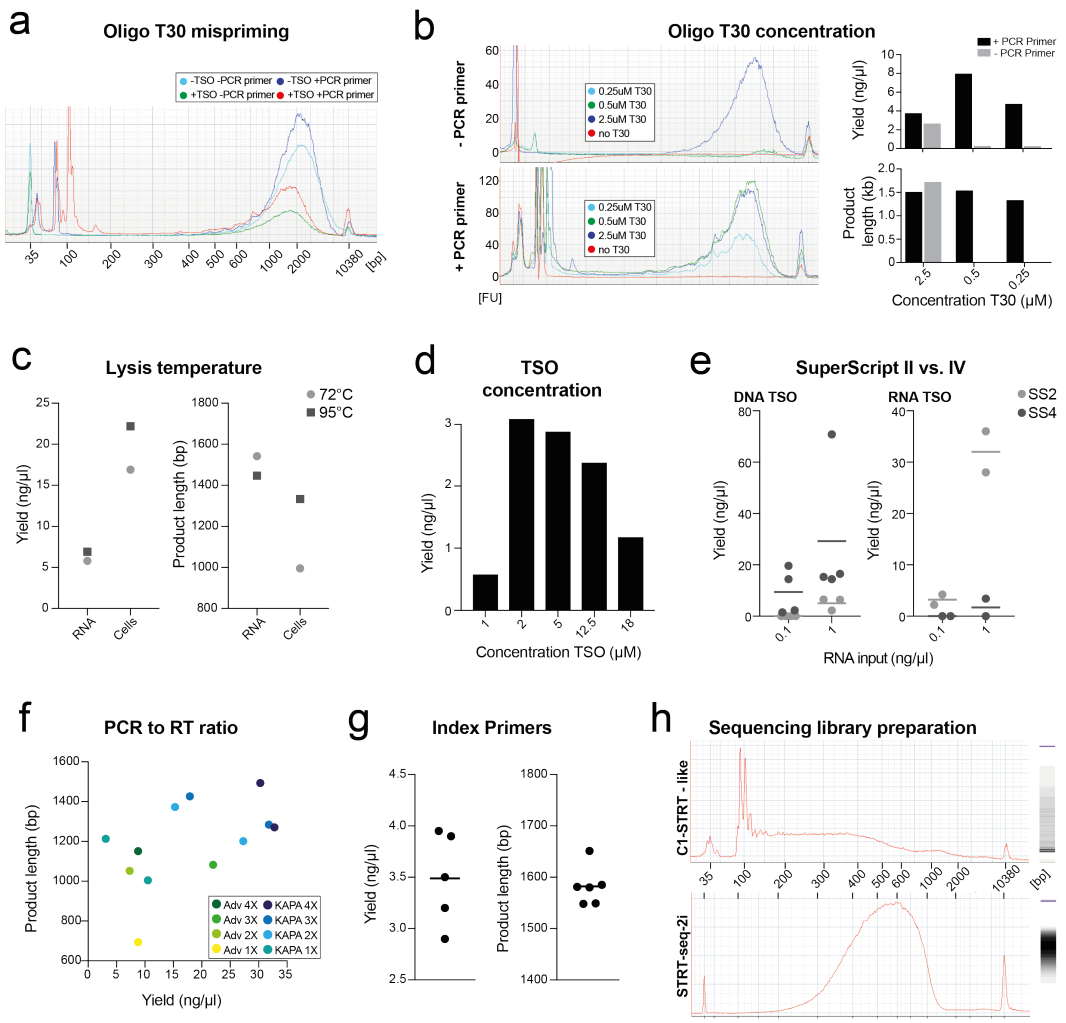

Figure S2

Examples of optimizations for STRT-seq-2i. (a) Bioanalyzer traces of test reactions omitting template switch oligo (TSO), PCR Primer or both. T30 oligo, intended to capture polyadenylated RNA molecules, was found to misprime both as TSO and PCR primer, which resulted in longer than usual libraries. (b) Bioanalyzer traces (left) and quantification (right) of decreasing T30 concentrations. Low T30 concentrations eliminated its mispriming activity in the absence of PCR primers, yet yielded suitable cDNA libraries in the presence of PCR primers. (c) Increasing lysis temperature from 72°C to 95°C increased cDNA yield from cells and improved library length. (d) Higher concentration of template switch oligo (TSO) gave better yield, but reached saturation at 5µM. (e) Despite faster reaction rate, reverse transcriptase SuperScript IV (SS4) was inferior to SuperScript II (SS2) using a RNA TSO. (f) Increasing the ratio of PCR:RT reaction volume yielded longer and more cDNA both with Advantage2 and KAPA HiFi polymerase. (g) Well-index primers with different barcodes performed comparably in PCR. (h) STRT-seq-2i sequencing library preparation was adapted to better match the desired size distribution for Illumina platforms, compared to the original C1-STRT protocol.

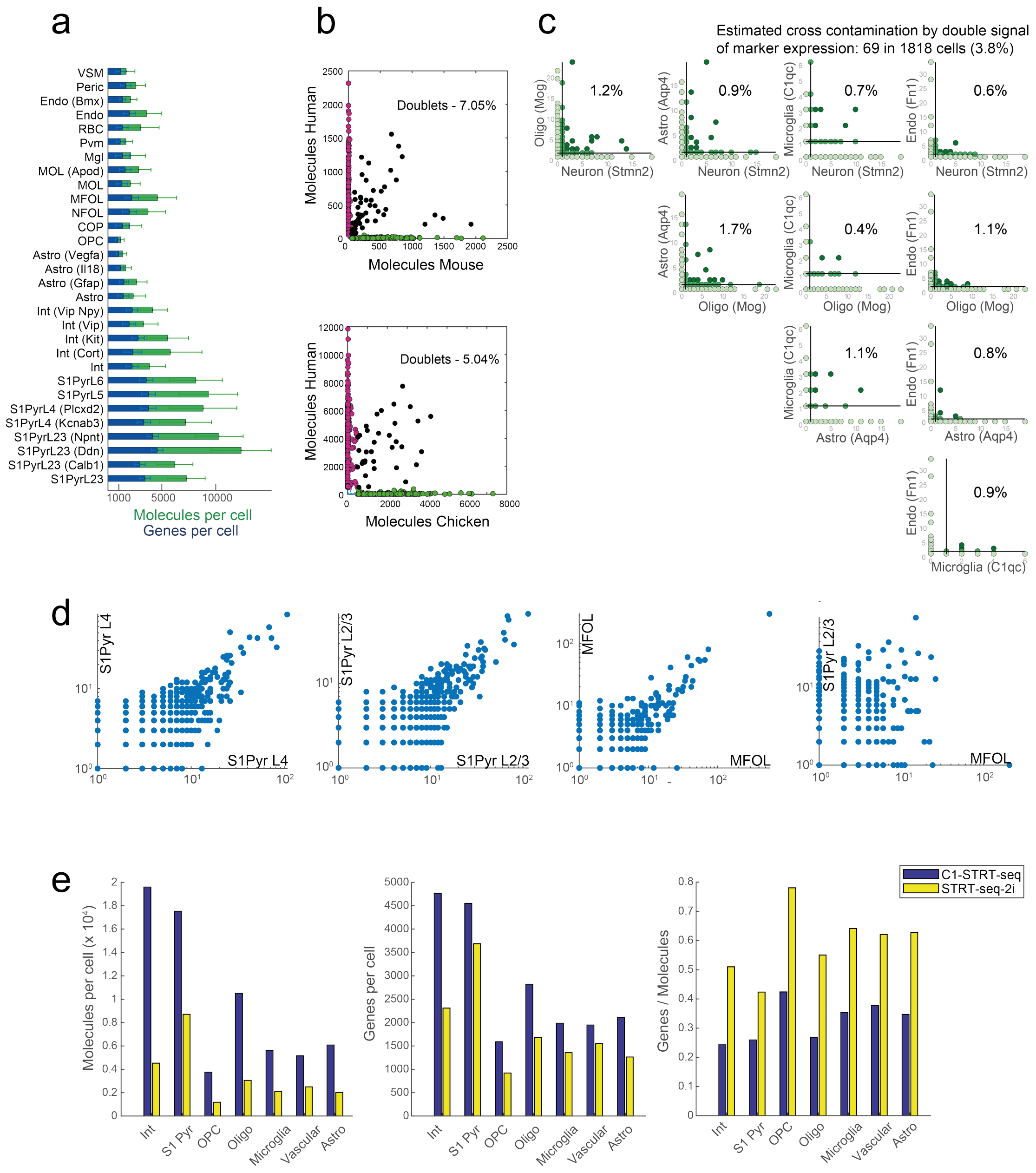

Figure S3

(a) Variation of detected number of molecules and genes per cell, depending on cortical cell type. (b) Doublet rates in two-species experiments (Fig. 1e and 1f visualized by species-specific molecule counts. (c) Estimated cross-contamination by double-expression of the main cell type-specific markers in cortex dataset. (d) Expression correlation scatterplot between cortex cells of the same cell type (plots 1-3) and two different cell types (plot 4). (e) Performance comparison C1-STRT-seq (Zeisel *et al.* 2015) and STRT-seq-2i, per mouse somatosensory cortex cell classes. Sequencing depth as mapped mRNA molecules was ~500,000 for C1-STRT-seq and ~40,000 for STRT-seq-2i.

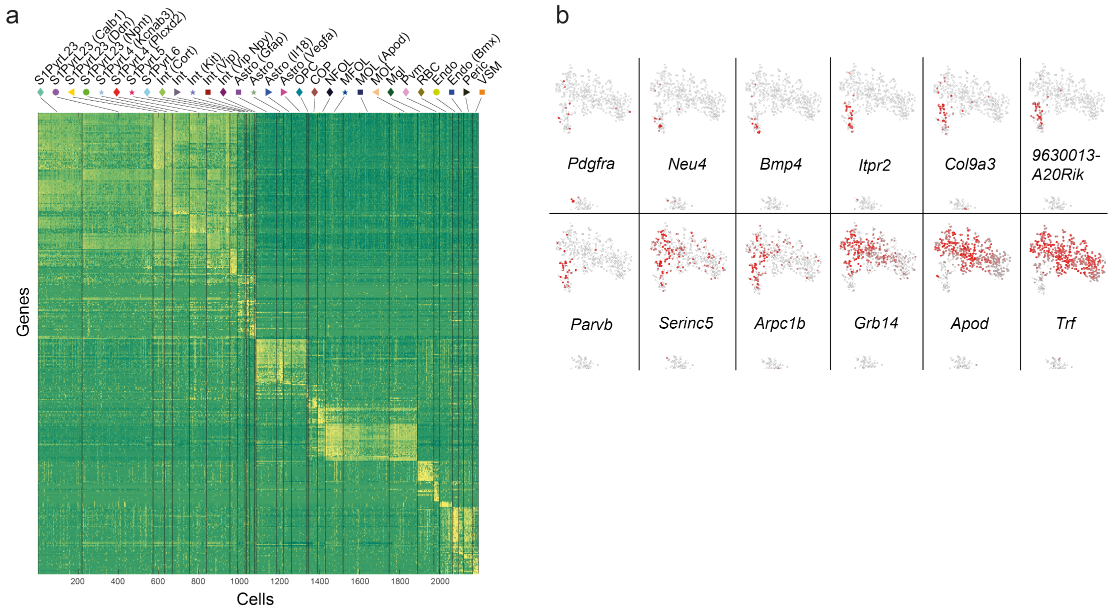

Figure S4

(a) Heatmap of clusters represented by their top marker genes, expression normalized by gene. (b) zoom-in of indicated area in Fig. 1(a) on cells of the oligodendrocyte lineage, indicating the expression of subtype-specific marker genes.

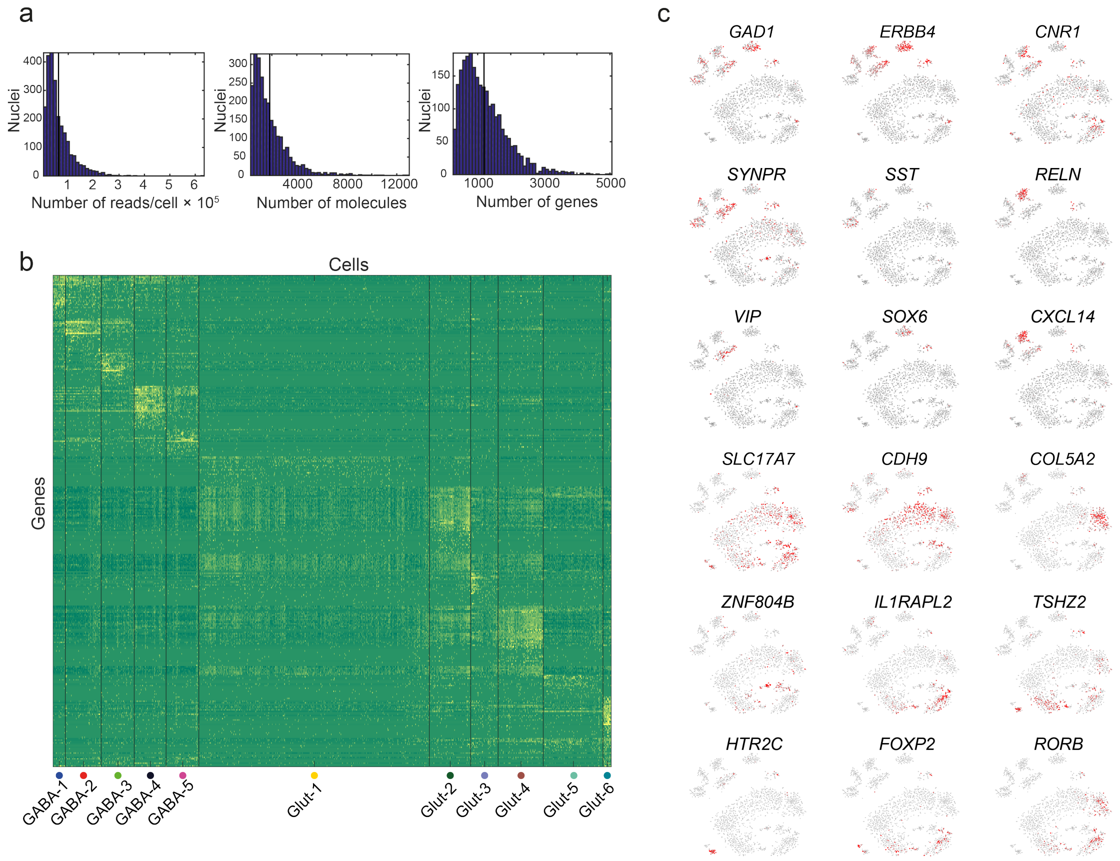

Figure S5

STRT-seq-2i on human post-mortem nuclei. (a) Distribution of the number of reads (mean 61785), detected molecules (mean 1828) and genes (mean 1192) per cell. (b) Heatmap of clusters represented by their top marker genes, expression normalized by gene. (c) tSNE visualisations of the nuclei, stained by subtype- or cluster-specific gene expression (rows 1-3, GABA; rows 4-6, Glut).

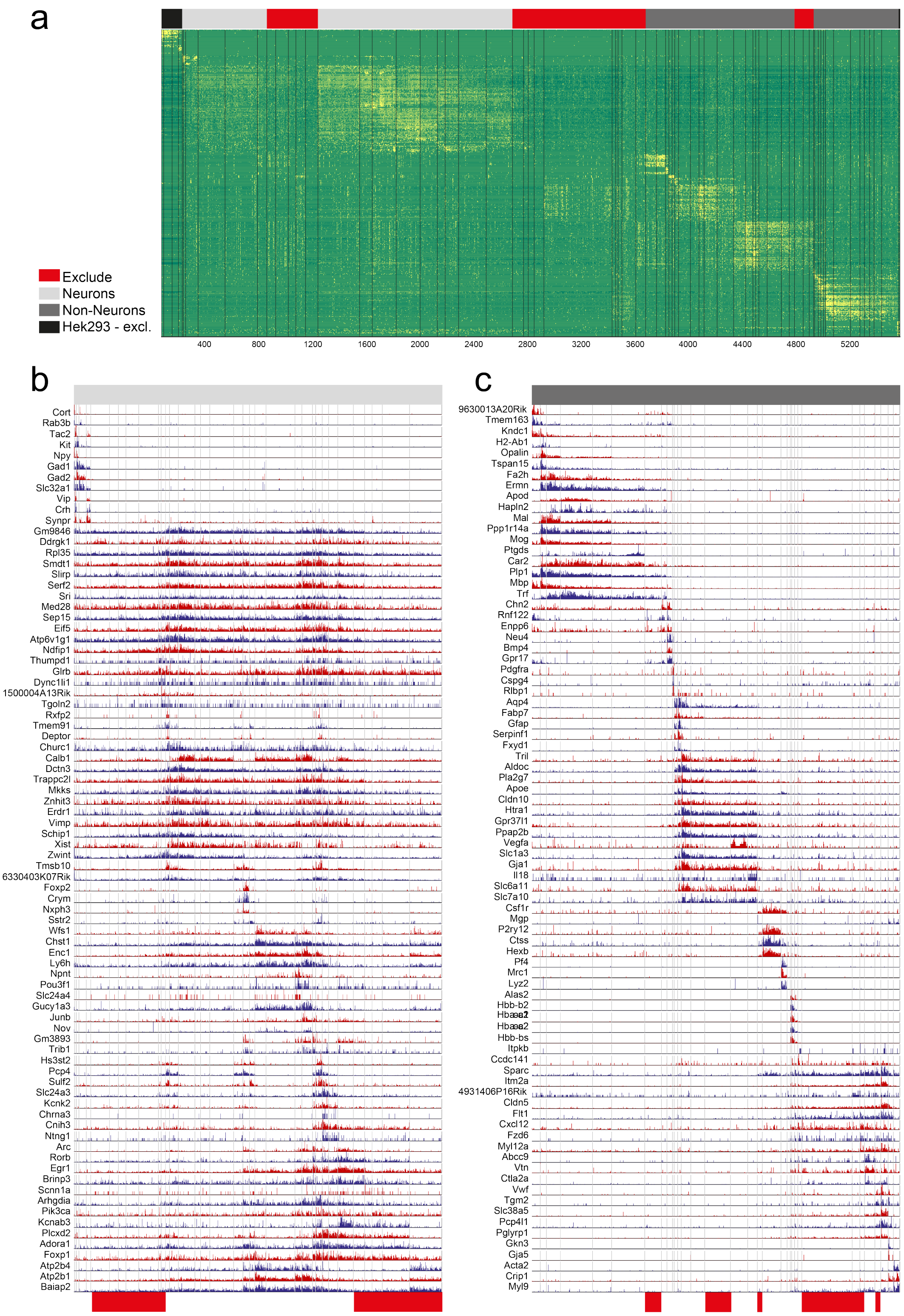

Figure S6

Overview of BackSPINv2 clusters excluded in cortex analysis (Fig. 2). (a) Heatmap of gene expression across all cells, categorized to Neurons, Non-Neurons, Hek293 (excluded) and Excluded (due to low quality). (b-c) Barplots of marker genes for neurons (b) and non-neurons (c), with clusters excluded from further analysis, due to lack of cluster-specific markers, indicated below.

Table S1 Summary of experiments with data included in this manuscript.

Supplementary Table 1: Experimental log

Figure	Experiment ID	Dispense to # wells	Single cell wells	Success rat	Poisson fit	Cell disp. blocks	Species	Strain	Cells	Age	Protocol	Comment
Figure 1h	WG960025A	620	300	0.48	NA	NA	Hs	NA	Hek293	NA	Alternative C	FACS to 200nl PBS
Figure 1h	WG960025B	620	534	0.86	NA	NA	Hs	NA	Hek293	NA	Alternative C	FACS to 50nl PBS
Figure 1i	WG960031	1604	591	0.37	NA	NA	Hs	NA	Hek293	NA	Alternative C	FACS checkerboard pattern to 32 wells/subarray
Figure 1d, 2, S3a	WG960011	9583	2120	0.22	0.6	96	Mm	CD-1	Ss cortex	p25	Alternative A	Attention: Remove subarrays dispensed with Hek293 cells (test) from analysis
Figure 1d, 2, S3a	WG960015	4791	869	0.18	0.49	48	Mm	CD-1	Ss cortex	p23	Alternative A	
Figure 1d, 2, S3a	WG960016	4791	1381	0.29	0.78	48	Mm	CD-1	Ss cortex	p28	Alternative A	
Figure 1d, 1g, 2,	WG960017	9583	2495	0.26	0.71	96	Mm	CD-1	Ss cortex	p30	Alternative A	
Figure 1b-d, 2, S	WG960021	9583	2736	0.29	0.78	96	Mm	CD-1	SS cortex	p37	Alternative A	
Figure S1b	WG960030B	NA	NA	NA	NA	NA	Hs	NA	Hek293	NA	Alternative A	test cDNA
Figure S1b	WG960030C	NA	NA	NA	NA	NA	Hs	NA	Hek293	NA	Alternative B	test cDNA
Figure S1c	WG960025	NA	NA	NA	NA	NA	Mm	CD-1	SS cortex	p21	Alternative C	test cDNA, FACS checkerboard pattern 32 wells/subarray, to PBS or lysis buffer
Figure 1e-f, S3b	WG960034	9583	2237	0.23	0.73	96	Hs / Gg / Mm	NA	DF-1, Hek293, mE	NA	Alternative B	2x 2 species experiment (Hs-Gg, Hs-Mm)
Figure 2, S5	WG960039	9583	2842	0.3	0.93	96	Hs	NA	NeuN+ middle tem	NA	Alternative A	

Table S2 Cell sorter BD Influx instrument configuration and gating strategy for sorting of single cells to wells.

<table>
<tr><td>Instrument Manufacturer:</td><td>BD</td></tr>
<tr><td>Instrument model:</td><td>BD Influx</td></tr>
<tr><td>Serial number:</td><td>X646500Q8001</td></tr>
<tr><td>Software:</td><td>BD FACS Sortware 1.2.0.142/Utopex 1.2.0.108</td></tr>
<tr><td>Firmware Version:</td><td>7.5.1.3.16</td></tr>
<tr><td>Instrument configuration:</td><td>
<table>
<tr><th>Laser</th><th>Detector/Filters</th></tr>
<tr><td>488 nm</td><td></td></tr>
<tr><td></td><td>Forward Scatter (FSC)</td></tr>
<tr><td></td><td>Side Scatter (SSC)</td></tr>
<tr><td></td><td>505LP -> 530/40</td></tr>
<tr><td></td><td>550LP -> 710/50</td></tr>
<tr><td>640 nm</td><td></td></tr>
<tr><td></td><td>650LP -> 670/30</td></tr>
<tr><td></td><td>700LP -> 720/40</td></tr>
<tr><td></td><td>740LP -> 750LP</td></tr>
<tr><td>355 nm</td><td></td></tr>
<tr><td></td><td>400LP -> 460/50</td></tr>
<tr><td></td><td>550LP -> 670/30</td></tr>
<tr><td>561 nm</td><td></td></tr>
<tr><td></td><td>561RB -> 570LP -> 585/29</td></tr>
<tr><td></td><td>600LP -> 610/20</td></tr>
<tr><td></td><td>645LP -> 670/30</td></tr>
<tr><td></td><td>685LP -> 710/50</td></tr>
<tr><td></td><td>740LP -> 750LP</td></tr>
<tr><td>405 nm</td><td></td></tr>
<tr><td></td><td>420LP -> 460/50</td></tr>
<tr><td></td><td>480LP -> 520/35</td></tr>
<tr><td></td><td>550LP -> 610/20</td></tr>
<tr><td></td><td>645LP -> 660/20</td></tr>
<tr><td></td><td>685LP -> 710/50</td></tr>
<tr><td></td><td>740LP -> 750LP</td></tr>
</table>
</td></tr>
<tr><td>Sorter setup:</td><td>Nozzle: 140 µm
Pressure: 10.0 PSI
Drop frequency: 16.7-17.0 kHz
Amplitude: ≈18-30
Purity mode: 1.0 Drop Single
• Phase mask: 10/16
• Extra coincidence: 4/16
Sample rate: ≈ 500-1.000 events/sec</td></tr>
<tr><td>Instrument calibration:</td><td>CS&T beads; manual adjustment</td></tr>
<tr><td>Gating strategy:</td><td>1. Population of cells based on FSC-H x SSC-H
2. singlets based on FSC-H x FSC-W
3. singlets based on FSC-H x FSC-A
4. One of the options:
a. Cell-Tracker Green positive (530/40[488nm])
b. Cell-Tracker Green positive (530/40[488nm]) and Propidium iodide negative (585/29[561nm]) population</td></tr>
</table>
